# Supplementary material for: Followership styles scrutinized: temporal consistency and relationships with job attitudes and self-efficacy
Source: PeerJ. 2023 Oct 27;11:e16135. doi: 10.7717/peerj.16135 (PMC10615031; doi:10.7717/peerj.16135)
Supplement: Supplemental Information 1 [file peerj-11-16135-s001.pdf]

Followership Styles Scrutinized: Temporal Consistency and Relationships with Job Attitudes and Self-efficacy.

Supplemental Material 1. Testing For Measurement Equivalence.

In this SM1 we describe the procedures that we followed to test for measurement equivalence (ME). Following Geiser (2020), we consecutively tested ME models that differ by the level of ME (i.e., various parameter equality constraints) for every latent variable. First, we tested for configural invariance that specified the same number of factors and the same factor loading pattern across time. Second, we also constrained the factor loadings to remain the same for a given observed variable in addition to configural invariance (weak invariance). Subsequently, we tested for strong invariance (strong ME), which additionally set the intercepts to remain the same across time for a given observed variable. Finally, the strict invariance model (strict ME) additionally determined the measurement error variance to remain the same across time for a given variable.

Study 1

Table 1

Measurement Equivalence of AE

Model	χ^2	df	p	$\chi^2\Delta$	dfΔ	p($\chi^2\Delta$)	RMSEA	CFI	SRMR	AIC	BIC
Configural ME	432.44	167	.00				.09	.84	.08	10,526	10,728
Weak ME	438.34	176	.00	14.90	9	.094	.09	.84	.08	10,514	10,687
Strong ME	450.93	185	.00	12.59	9	.182	.09	.84	.08	10,509	10,653
Strict ME	476.55	195	.00	25.62	10	.005	.09	.83	.11	10,515	10,626

Note. *N* = 184. RMSEA = Root Mean Square Error of Approximation; CFI = Comparative Fit Index; SRMR = Standardized Root Mean Square Residual; AIC = Akaike's Information Criterion; BIC = Bayesian Information Criterion.

Table 2

Measurement Equivalence of ICT

Model	χ^2	df	p	$\chi^2\Delta$	dfΔ	p($\chi^2\Delta$)	RMSEA	CFI	SRMR	AIC	BIC
Configural ME	74.85	19	.00				.13	.80	.07	4,677	4,757
Weak ME	75.80	22	.00	0.95	3	.813	.12	.81	.08	4,672	4,742
Strong ME	76.44	25	.00	0.64	3	.887	.11	.82	.08	4,666	4,727
Strict ME	80.11	29	.00	3.67	4	.453	.10	.82	.09	4,662	4,710

Note. *N* = 184. RMSEA = Root Mean Square Error of Approximation; CFI = Comparative Fit Index; SRMR = Standardized Root Mean Square Residual; AIC = Akaike's Information Criterion; BIC = Bayesian Information Criterion.

Table 3

Measurement Equivalence of Job Satisfaction

Model	χ^2	df	p	$\chi^2\Delta$	dfΔ	p($\chi^2\Delta$)	RMSEA	CFI	SRMR	AIC	BIC
Configural ME	335.89	53	.00				.17	.80	.08	6,668	6,787
Weak ME	339.69	58	.00	3.80	5	.579	.16	.80	.08	6,662	6,764
Strong ME	344.43	63	.00	4.74	5	.448	.16	.80	.08	6,657	6,743
Strict ME	350.75	69	.00	6.32	6	.388	.15	.80	.08	6,651	6,718

Note. *N* = 184. RMSEA = Root Mean Square Error of Approximation; CFI = Comparative Fit Index; SRMR = Standardized Root Mean Square Residual; AIC = Akaike's Information Criterion; BIC = Bayesian Information Criterion.

Table 4

Measurement Equivalence of Organizational Commitment

Model	χ^2	df	p	$\chi^2\Delta$	dfΔ	p($\chi^2\Delta$)	RMSEA	CFI	SRMR	AIC	BIC
Configural ME	51.54	8	.00				.17	.90	.04	3,530	3,591
Weak ME	52.40	10	.00	0.86	2	.651	.15	.91	.05	3,527	3,581
Strong ME	55.90	12	.00	3.50	2	.174	.14	.90	.05	3,526	3,574
Strict ME	66.46	15	.00	10.56	3	.014	.14	.89	.08	3,531	3,569

Note. *N* = 184. RMSEA = Root Mean Square Error of Approximation; CFI = Comparative Fit Index; SRMR = Standardized Root Mean Square Residual; AIC = Akaike's Information Criterion; BIC = Bayesian Information Criterion.

Table 5

Measurement Equivalence of Self-Efficacy

Model	χ^2	df	p	$\chi^2\Delta$	dfΔ	p($\chi^2\Delta$)	RMSEA	CFI	SRMR	AIC	BIC
Configural ME	309.53	53	.00				.16	.80	.07	5,892	6,010
Weak ME	311.50	58	.00	1.97	5	.853	.16	.80	.08	5,884	5,986
Strong ME	314.73	63	.00	3.23	5	.665	.15	.80	.08	5,877	5,963
Strict ME	332.55	69	.00	17.82	6	.007	.15	.80	.12	5,883	5,950

Note. *N* = 184. RMSEA = Root Mean Square Error of Approximation; CFI = Comparative Fit Index; SRMR = Standardized Root Mean Square Residual; AIC = Akaike's Information Criterion; BIC = Bayesian Information Criterion.

Study 2

Table 6

Measurement Equivalence of AE

Model	χ^2	df	p	$\chi^2\Delta$	dfΔ	p($\chi^2\Delta$)	RMSEA	CFI	SRMR	AIC	BIC
Configural ME	1345.4	167	.00				.11	.83	.06	32,728	33,000
Weak ME	1354.5	176	.00	9.1	9	.428	.11	.83	.07	32,719	32,953
Strong ME	1363.1	185	.00	8.6	9	.475	.11	.83	.07	32,710	32,904
Strict ME	1370,0	195	.00	6.9	10	.735	.10	.83	.07	32,696	32,848

Note. *N* = 570. RMSEA = Root Mean Square Error of Approximation; CFI = Comparative Fit Index; SRMR = Standardized Root Mean Square Residual; AIC = Akaike's Information Criterion; BIC = Bayesian Information Criterion.

Table 7

Measurement Equivalence of ICT

Model	χ^2	df	p	$\chi^2\Delta$	dfΔ	p($\chi^2\Delta$)	RMSEA	CFI	SRMR	AIC	BIC
Configural ME	179.67	19	.00				.12	.87	.05	14,396	14,504
Weak ME	180.79	22	.00	1.12	3	.772	.11	.87	.05	14,391	14,486
Strong ME	184.09	25	.00	3.30	3	.348	.11	.87	.06	14,388	14,470
Strict ME	184.83	29	.00	0.74	4	.946	.10	.87	.06	14,381	14,446

Note. *N* = 570. RMSEA = Root Mean Square Error of Approximation; CFI = Comparative Fit Index; SRMR = Standardized Root Mean Square Residual; AIC = Akaike's Information Criterion; BIC = Bayesian Information Criterion.

Table 8

Measurement Equivalence of Job Satisfaction

Model	χ^2	df	p	$\chi^2\Delta$	dfΔ	p($\chi^2\Delta$)	RMSEA	CFI	SRMR	AIC	BIC
Configural ME	904.15	53	.00				.17	.83	.06	18,485	18,645
Weak ME	906.57	58	.00	2.42	5	.789	.16	.83	.06	18,478	18,615
Strong ME	911.56	63	.00	4.99	5	.417	.16	.83	.06	18,473	18,589
Strict ME	917.24	69	.00	5.68	6	.460	.15	.83	.06	18,466	18,557

Note. *N* = 570. RMSEA = Root Mean Square Error of Approximation; CFI = Comparative Fit Index; SRMR = Standardized Root Mean Square Residual; AIC = Akaike's Information Criterion; BIC = Bayesian Information Criterion.

Table 9

Measurement Equivalence of Organizational Commitment

Model	χ^2	df	p	$\chi^2\Delta$	dfΔ	p($\chi^2\Delta$)	RMSEA	CFI	SRMR	AIC	BIC
Configural ME	216.43	8	.00				.22	.88	.06	10,832	10,913
Weak ME	216.86	10	.00	0.43	2	.807	.19	.89	.06	10,828	10,901
Strong ME	220.67	12	.00	3.81	2	.149	.18	.88	.06	10,828	10,892
Strict ME	222.34	15	.00	1.67	3	.644	.16	.89	.06	10,823	10,875

Note. *N* = 570. RMSEA = Root Mean Square Error of Approximation; CFI = Comparative Fit Index; SRMR = Standardized Root Mean Square Residual; AIC = Akaike's Information Criterion; BIC = Bayesian Information Criterion.

Table 10

Measurement Equivalence of Self-Efficacy

Model	χ^2	df	p	$\chi^2\Delta$	dfΔ	p($\chi^2\Delta$)	RMSEA	CFI	SRMR	AIC	BIC
Configural ME	419.20	53	.00				.11	.94	.04	16,123	16,282
Weak ME	421.26	58	.00	2.06	5	.841	.11	.94	.04	16,115	16,253
Strong ME	428.26	63	.00	7.00	5	.221	.10	.94	.04	16,112	16,228
Strict ME	442.40	69	.00	14.14	6	.028	.10	.93	.05	16,114	16,204

Note. *N* = 570. RMSEA = Root Mean Square Error of Approximation; CFI = Comparative Fit Index; SRMR = Standardized Root Mean Square Residual; AIC = Akaike's Information Criterion; BIC = Bayesian Information Criterion.

Reference:

Geiser, C. (2020). *Longitudinal structural equation modeling with Mplus. A latent state-trait perspective*. Guilford.
