# Supplementary material for: Followership styles scrutinized: temporal consistency and relationships with job attitudes and self-efficacy
Source: PeerJ. 2023 Oct 27;11:e16135. doi: 10.7717/peerj.16135 (PMC10615031; doi:10.7717/peerj.16135)
Supplement: Supplemental Information 2 [file peerj-11-16135-s002.pdf]

Followership Styles Scrutinized: Temporal Consistency and Relationships with Job Attitudes and Self-efficacy.

Supplemental Material 2. Distribution of AE and ICT mean values (at t1).

The distributions of the mean values for both followership scales were as follows:

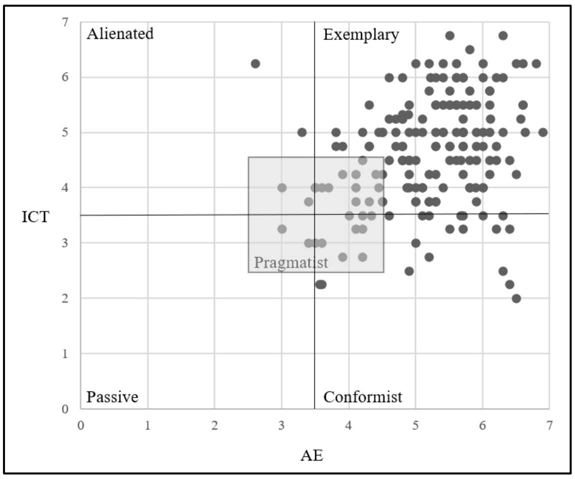

Distribution of AE and ICT mean values for Study 1 (t1)

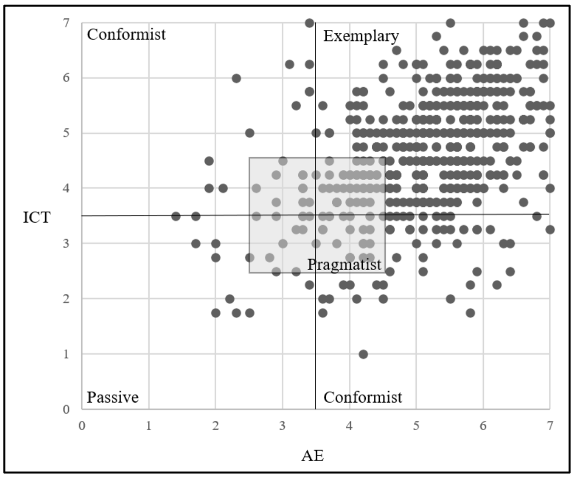

Distribution of AE and ICT mean values for Study 2 (t1)

We see that most participants in both studies adopted the pragmatist or exemplary followership style.
